# Do wearing masks and preservatives have a combined effect on skin health?

**DOI:** 10.1016/j.eehl.2024.01.003

**Published:** 2024-01-24

**Authors:** Yu Liu, Leijian Chen, Shuyi Zhang, Xiaoxiao Wang, Yuanyuan Song, Hongwen Sun, Zongwei Cai, Lei Wang

**Affiliations:** a MOE Key Laboratory of Pollution Processes and Environmental Criteria, College of Environmental Science and Engineering, Nankai University, Tianjin 300350, China; b State Key Laboratory of Environmental and Biological Analysis, Department of Chemistry, Hong Kong Baptist University, Hong Kong 999077, China

**Keywords:** MeP, Hypoxia, Synergistic effect, Skin metabolomics, Network toxicology

## Abstract

Chemical exposure and local hypoxia caused by mask-wearing may result in skin physiology changes. The effects of methylparaben (MeP), a commonly used preservative in personal care products, and hypoxia on skin health were investigated by HaCaT cell and ICR mouse experiments. MeP exposure resulted in lipid peroxidation and interfered with cellular glutathione metabolism, while hypoxia treatment disturbed phenylalanine, tyrosine, and tryptophan biosynthesis pathways and energy metabolism to respond to oxidative stress. A hypoxic environment increased the perturbation of MeP on the purine metabolism in HaCaT cells, resulting in increased expression of proinflammatory cytokines. The synergistic effects were further validated in a mouse model with MeP dermal exposure and “mask-wearing” treatment. CAT, PPARG, and MMP2 were identified as possible key gene targets associated with skin health risks posed by MeP and hypoxia. Network toxicity analysis suggested a synergistic effect, indicating the risk of skin inflammation and skin barrier aging.

## 1 Introduction

The largest organ in the body is the skin. Many chemicals could be absorbed into the human body via the skin, which may negatively affect skin health [1]. Parabens, aliphatic esters derived from p-hydroxybenzoic acid, are extensively applied as antimicrobial ingredients in the storage of maquillage [2], and the mostly used parabens are methylparaben (p-hydroxybenzoic acid methyl ester, MeP) [2]. In the Czech Republic study, MeP was detected in 72% of personal care products (n = 53), and the maximum measured concentration was up to 4,522 μg/g [3]. When the MeP-containing product is used frequently, MeP could be retained and accumulate in the stratum corneum without hydrolysis [4]. As an endocrine-disrupting chemical, the adverse skin health effects of parabens have gradually been reported [5,6]. MeP could influence skin aging and induce oxidative stress in rat skin [5], and higher concentrations (0.3%, W/V) of MeP caused a significant increase in necrotic cells [6].

The human epidermis directly utilizes oxygen from the atmosphere [7]. Under a hypoxia environment, the migration of keratinocytes could be inhibited, melanin production could be promoted, and human skin sensitivity to temperature stimuli could be reduced [[8], [9], [10]]. Wearing a personal protective mask might form a closed and hypoxic environment on the surface of the facial skin [11], which might lead to changes in glycerophospholipid metabolism and sphingolipid metabolism of human skin [12]. Furthermore, the epidermal thickness and proliferation were reduced under hypoxia conditions for 3 days [13], which might cause combined effects on skin health and other factors. For example, a mild hypoxic environment increased UVB-induced apoptosis of normal human epidermal keratinocytes [14]. In practice, personal care products and masks are often used concurrently. Due to the emphasis on air pollution and the influence of the COVID-19 pandemic, the frequency of mask use has significantly increased on many occasions [15]. During the pandemic, 65.9% (n = 164) of participants maintained skin care habits [16]. The use of personal care products containing paraben increased with the increasing frequency of skin cleaning. It became more likely that hypoxia and parabens would be present simultaneously. Previous research suggested EPA6 transcription could be stimulated by hypoxic and paraben exposure and resulting in enhanced adherence to vaginal epithelial cells *in vitro* [17]. Nevertheless, the possible cumulative effect of parabens and hypoxia on skin health remains uncertain.

Metabolomics could offer a comprehensive and detailed understanding of the internal metabolic condition of organisms, including pathogenic or toxicological occurrences from a holistic metabolic standpoint, providing crucial information for analyzing the toxic mechanisms of chemicals [18]. Skin metabolomics has been applied to characterize physiological skin functions and to discover metabolic changes due to chemical treatment or environmental changes [12,19]. Network toxicology can clarify the relationships between chemicals and the human body by considering the broader context of a biological network, which can elucidate the mechanism underlying adverse reactions of chemicals and highlight the complex regulation of signal pathways across multiple channels [20]. The metabolomics and network toxicology method could improve the accuracy and effectiveness [21,22].

In this study, *in vitro* experiments combining network toxicology methodology with untargeted metabolomics were conducted to explore how MeP and/or hypoxia cause dermal toxicity. Furthermore, a mouse model validated the combined skin effects caused by MeP and “mask-wearing”. Based on the results of *in vitro* and *in vivo* experiments, the biomarkers of MeP and/or hypoxia-induced skin toxicity were detected, the combined effect of the two treatments was identified, and the mechanism of adverse reactions was explained.

## 2 Materials and methods

### 2.1 Regents and materials

Standards of MeP were obtained from J&K Scientific with a purity of 98% (China). The following items were obtained from Gibco (Thermo Fisher Scientific, Waltham, MA): Penicillin–streptomycin, Fetal bovine serum (FBS), phosphate-buffered saline (PBS), 0.25% trypsin–EDTA and Dulbecco's Modified Eagle Medium (DMEM) high glucose cell culture medium. Formic acid (FA), acetonitrile (ACN), and Methanol (MeOH) were HPLC grade and purchased from Sigma-Aldrich (Shanghai, China). Dimethylsulfoxide (DMSO) and 4-chloro-phenylalanine (4-Cl-Phe) were purchased from Sigma-Aldrich (St. Louis, MO, USA).

### 2.2 Cell culture

Human immortal keratinocyte cell line HaCaT was acquired from the Procell Life Science & Technology Co., Ltd. HaCaT cells were cultivated in DMEM medium with 1% penicillin/streptomycin and 10% FBS. Cells were maintained in a 5% CO_2_ atmosphere at 37 °C.

### 2.3 Experimental animal

ICR mice (Male, 7-week-old) were obtained from the Chinese University of Hong Kong. The Hong Kong Department of Health granted approval for all animal testing procedures and followed the standards for animal care and use published by the National Institutes of Health in the United States. Mice are kept in ventilated and temperature-controlled animal rooms (temperature 20 ± 2 °C, relative humidity 60% ± 10%, 12 h/dark cycle), with food and water freely available.

### 2.4 Mice experiment design

Before the official start of the experiment, the hair on the back of the mice was scraped off to expose the skin tissue and adapted for 1–2 weeks, and the health status was monitored daily. At the beginning of the experiment, mice were randomly assigned into 4 groups, with 6 mice per group. Breathable adhesive tape with a 5 mg/kg MeP cotton sheet was pasted on the hairless area on the back of mice to serve as the MeP group. Non-breathable adhesive tape to simulate a mask was attached to the mice's back to serve as a mask group. For the mask + MeP group, the back of the mice was covered with a non-breathable adhesive tape containing MeP. We fixed the cotton pads on the exposed skin on the back of the mice for 48 h, anesthetized the mice with isoflurane, then collected skin samples from the administration site using D-squame tap strips and stored them at −80 °C until further analysis. The experimental design of this part is illustrated in Fig. S1.

### 2.5 Cell viability assay

A 2 × 10^4^/200 mL medium density of HaCaT cells were seeded in 96-well plates and incubated for 24 h. The original medium was substituted with serum-free medium containing serial concentrations of MeP (0, 1, 10, 50, 100, 200, 300, 400, 500, 800, 1000 μM) dissolved in DMSO (0.05%, v/v) and/or hypoxic environment for a 48-h exposure. The oxygen content of air inside the KN95 mask was 16.8% ± 0.56% [11]. Thus, the cell culture dish was placed in a hypoxic bag for hypoxic treatment, and nitrogen gas was slowly added until the oxygen content inside the bag dropped to 16.0%. We then closed the bag and placed it into the 37 °C incubator. A gas detector (Honeyeagle, Shenzhen, China) was used to test the gas concentration in the closed bag during the treatment period every 6 h to ensure that the oxygen ratio was 16.0% ± 0.5%. After MeP and/or hypoxia treatment, cell viability was evaluated by a commercial Cell Counting Kit-8 assay (Dojindo, Japan). The dose level of the control was 0 μM, and the blank group consisted solely of a medium devoid of cells. After these treatments, the microplate reader was used to measure the absorbance of cells at 450 nm.

### 2.6 Cell metabolomic analysis

Initially, cells (a total of 1.25 × 10^6^) were placed in 6 cm culture dishes containing a complete medium for 24 h. Subsequently, the cells were cultivated in a serum-free medium, as determined by the MeP cell viability findings (0, 500, 800 μM) and/or a hypoxic environment for another 48 h. Each group was provided with eight replicates to quantify metabolomics. After 48 h exposure, the culture medium was extracted, and the cells were immediately rinsed with PBS on two occasions. Next, the cells were quickly cooled down by adding 500 μL of chilled MeOH/H_2_O (4:1, v/v) and collected using scrapers into 2.0 mL Eppendorf tubes. Cells were lysed by subjecting them to five freeze–thaw cycles using liquid nitrogen. The cell suspensions that were disturbed were subjected to centrifugation at a speed of 15,000*g* for a duration of 10 min at a temperature of 4 °C. The supernatants were gathered and subjected to evaporation until completely dried using a controlled flow of nitrogen gas. A solution consisting of 50% methanol (MeOH) and 50% water (H_2_O), containing 1 microgram per milliliter (μg/mL) of 4-Cl-Phe [the internal standard (IS)], was utilized to dissolve the remaining substances. The supernatants were collected after sample centrifugation (15,000*g*, 10 min, 4 °C). The quality control (QC) sample was made by amalgamating 20 μL solution from each of the samples.

### 2.7 Biological measurements associated with pathways

Xanthine oxidase (XOD) activity associated with purine metabolism and the activity of glutathione peroxidase (GSH-Px) linked with glutathione (GSH) metabolism were investigated. Cell lysis buffer was used to harvest MeP and/or hypoxia-treated cells, and the supernatants were gathered after centrifugating for 5 min (15,000*g*, 4 °C). GSH-Px and XOD assay kits from Beyotime Institute of Biotechnology (Beijing, China) and Nanjing Jiancheng Bioengineering Institute (China), were utilized to determine their activities under the guidelines provided by the manufacturers.

### 2.8 Detection of ROS and oxidative stress indicators

To determine the effect of MeP and hypoxia environment on the production of reactive oxygen species (ROS) by HaCaT, HaCaT were cultured in the culture flask in the middle depression of the confocal small dish and grown in complete media until merged. After adhering to the wall, HaCaT was treated with 0, 500, 800 μM MeP and/or hypoxia for 48 h. HaCaT were then stained with 10 μM dihydroethidium (DHE; Sigma-Aldrich Corp) and incubated for 30 min (37 °C). After washing with PBS, cells were photographed using a laser scanning confocal microscope (LSM880 with Airyscan, Zeiss, Germany) with the same standardized settings for intensity.

Assay kits (Beyotime Institute of Biotechnology, Beijing, China) were applied to measure the malondialdehyde (MDA) level, catalase (CAT), and superoxide dismutase (SOD) activity in HaCaT cells. The assays were conducted according to the manufacturer's instructions. All results were standardized by protein concentrations.

A quantity of 0.1 g skin tissue was mixed with a phosphate buffer to make a homogenate solution. The ROS and MDA levels in mice's skin were measured according to the kit instructions.

### 2.9 mRNA levels of proinflammatory factor associated with HaCaT cells

HaCaT cells were seeded in 6-well plates with the complete medium (1.25 × 10^6^ cells/well) and cultured statically for 24 h. Except for the control group, 500, 800 μM MeP was added to induce HaCaT cells in other wells in the normal environment or hypoxic environment. After 48 h treatment, cells were washed twice with PBS. Total RNA was extracted from cells using the Trizol reagent (Life Technologies). The RT-PCR was used to guide the reverse transcription process. A kit (2× M5 HiPer SYBR Premix Es Taq) was used. The cDNA was amplified with GAPDH as an internal reference and TNF-α, 1L-1β, and 1L-8 as primers. The primer sequences and amplification conditions are listed in Table S1.

### 2.10 Mice metabolomic analysis

The extraction of metabolites from the mice skin for analysis was achieved using a modified methodology described in a previous study [12]. The thawed mice skin samples were briefly spiked with 500 μL of MeOH containing 0.5 μg/mL of IS (4-Cl-Phe). Then, 10 mL of MeOH was added as an extractant. The solution was agitated for 60 s using a vortex mixer and then centrifuged for 15 min (12,000*g*, 4 °C). A Max-Up (NB-504CIR) IR vacuum concentrator (N-Biotek Inc., GyeongGi-Do, Korea) was used to dry the supernatant at 4 °C. A mixture of 50:50 (v/v) methanol and water was used to reconstitute the residues. Prior to testing, the mixture underwent another round of centrifugation at 21,000*g*, 4 °C, for 15 min.

### 2.11 Gene targets identification and molecular docking

GeneCards (https://www.genecards.org/), Online Mendelian Inheritance in Man (OMIM) database (https://omim.org/), the Comparative Toxicogenomics Database (http://ctdbase.org/) and Swiss Target Prediction database (http://www.swisstargetprediction.ch/) were utilized to ascertain chemical and health risk targets. The “Chemical-target-risk” network was created by Cytoscape 3.9.1 software (http://cytoscape.org). The protein–protein interaction (PPI) data was acquired from the STRING database (https://string-db.org/), which was subsequently imported into Cytoscape 3.9.1 for visualization of the PPI network.

ChemBio Ultra 14.0 software (PerkinElmer, Waltham, MA, USA) was used to generate the two-dimensional structure of MeP. To optimize compound space conformations and decrease its energy, Chem3D Pro 14.0 software (PerkinElmer) was used. From the PDB database (http://www1.rcsb.org/), the 3D structures of essential proteins were retrieved. The Autodock 1.5.7 software (https://autodock.scripps.edu/) was utilized to determine the binding modes and interaction energies between MeP and crucial proteins. The lowest binding energy models were identified by scanning the binding sites. Visualization was done with PyMOL 2.5 software (https://pymol.org/2/).

### 2.12 Instrumental analysis for metabolomic study

An ultra-high-performance liquid chromatography-orbitrap high-resolution mass spectrometry (UHPLC-Orbitrap-HRMS) (Thermo Fisher Scientific, Waltham, MA) was used for metabolomics analyses. The column was Waters ACQUITY UPLC HSS T3 column (100 × 2.1 mm, 1.8 μm). The precise conditions for the chromatography were optimized under the findings of a prior study [12]. A full scan mode was employed for untargeted metabolite profiling, with parallel reaction monitoring for MS^2^ fragments. The chromatographic and MS parameters were meticulously documented in Table S2.

### 2.13 Metabolomics data processing

Xcalibur software (Thermo Scientific, San Jose, CA) was used to analyze the metabolomics data, and the XCMS package in R was used to extract the peak [23]. To remove interference, the metabolic characteristics of QC samples with a standard deviation greater than 30 % relative and disrupted signals in the blanks were removed. For the remaining qualified data, partial least squares with discriminant analysis (PLS-DA) and volcano plots using SIMCA 14.1 (Umetrics, Umea, Sweden) were utilized. Both *p* < 0.05 and fold change (FC) < 0.8 or > 1.2 were considered as the two criteria for identifying metabolic features that set the control group apart from the treatment groups.

To identify metabolites, the MS^2^ data were subjected to additional processing using Compound Discovery software (Thermo Scientific). The procedure entailed comparing the MS^2^ spectra of the metabolites with the retention time, accurate precursor mass, and isotope pattern from the database. The Human Metabolome Database's (https://hmdb.ca/) open-access metabolic databases and the mzCloud library were the metabolite libraries that were used. The allowable deviation in mass for both precursor and fragment ions was defined as 10 parts per million (ppm). Adduct ions of [M+Na]^+^, [M+NH_4_]^+^, and [M+H]^+^ were searched in positive ionization mode; meanwhile, [M+HCOO]^−^ and [M−H]^−^ were selected for negative ionization mode. MetaboAnalyst 5.0 (https://www.metaboanalyst.ca/) was used to analyze metabolic pathways in compliance with the Kyoto Encyclopedia of Genes and Genome.

### 2.14 Statistical analysis

The SPSS 24.0 software (IBM, USA) was used to analyze statistical differences between groups using either a one-way or two-way analysis of variance. The homogeneity of variance ascertained by Levene's test was the basis for performing either the Bonferroni (homoscedasticity) or Dunnett T3 (heteroscedasticity) post hoc tests. Statistical significance was defined as *p* < 0.05.

## 3 Results and discussion

### 3.1 Cell viability and metabolomics changes induced by MeP exposure

High levels of MeP exposure resulted in the inhibition of cell viabilities (Fig. S2). At exposure concentrations of 0–400 μM, no significant change in cell viability of HaCaT keratinocyte was caused by the exposure to MeP (Fig. S2). However, when exposed to MeP of 500–1,000 μM, the cell viability decreased to 58.3%–91.1%.

To unveil the degree of oxidative stress, MeP treatment of 500 μM and 800 μM that inhibited 10% and 20% cell viability was selected. MeP exposure induced ROS production (Fig. S3), resulting in oxidative stress and a potential pro-oxidative environment in HaCaT cells. The SOD-catalyzed O_2_^−^ disproportionation results in the production of H_2_O_2_, which is subsequently reduced to H_2_O by CAT. The exposure to 800 μM-MeP resulted in increased activities of SOD (Fig. S4A), whereas the activities of CAT reduced dramatically (Fig. S4B), leading to the buildup of ROS within the cells. Furthermore, the levels of MDA significantly increased after MeP treatment (Fig. S4C), which was direct evidence that MeP may cause lipid peroxidation in normal keratinocytes.

**Fig. 1:**
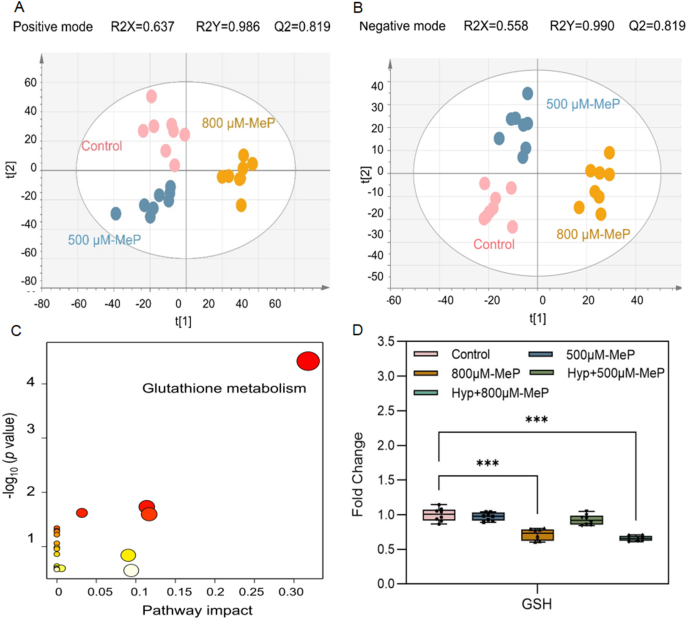
Three-dimensional PLS-DA score plots of HaCaT extract of the control treatment and the MeP treatment of 500 and 800 μM in positive (A) and negative (B) modes. The quality parameters, R2Y and Q2, are significantly greater than 0.5, indicating that the PLS-DA model has good fitting and predictive ability. (C) Metabolic pathway enrichment identified among differentially expressed metabolites in the MeP-treated HaCaT cell. (D) Fold change of GSH in HaCaT cells of different treatments. ∗∗∗*p* < 0.001.

The global MS-based untargeted metabolomics approach can measure the specific interference pathways in response to pollutant exposure [24]. The partial least squares discriminant analysis (PLS-DA) models indicated that the distribution of metabolites in the HaCaT cells exposed to MeP could be separated from those in the control treatment in both the positive and negative modes (Fig. 1A and B). A total of 15 endogenous differential metabolites (*p* < 0.05, FC values > 1.2 or < 0.8) induced by MeP exposure were identified in the HaCaT cell line by searching mass spectral libraries after MS^2^ spectra matching (Tables S3 and S4). These metabolites could be classified into organic acids and derivatives (6/15), lipids and lipid-like molecules (4/15), nucleosides, nucleotides, and analogs (3/15), organic oxygen compounds (1/15), and benzenoids (1/15), at the super-class level. The intracellular metabolism disturbances caused by MeP exposure showed a dose-dependent pattern (Fig. S5).

Analysis using MetaboAnalyst (5.0) based on the KEGG database indicated that the perturbance of GSH metabolism (pathway impact > 0.05 and Holm adjust *p* < 0.05) in HaCaT cells was identified to be highly associated with MeP exposure (Fig. 1C). GSH is a critical player in maintaining antioxidant defense systems inside the cell [25]. The abundance of GSH significantly decreased in the 800 μM-MeP group, with an FC of 0.658 (Fig. 1D). Significantly higher ROS signals were observed in 800 μM-MeP-treated HaCaT cells than in controls (Fig. S3). The increased consumption of GSH was an adaptive response to the overproduction of ROS [26]. In biological systems, moderate oxidation can result in the formation of oxidized glutathione (GSSG), and GSH/GSSG is a reliable indicator of cellular redox status. GSH/GSSG was significantly decreased with an FC of 0.633 in the 800 μM-MeP treatment (Fig. S6), reflecting a reduced antioxidant capacity and increased vulnerability to oxidative damage [27]. Consistently, the activity of GSH-Px, which catalyzes the oxidation of GSH to GSSG for reducing ROS, increased (Fig. S7). Due to the decrease in GSH levels, cells may maintain sufficient reduction equivalents by increasing glutathione reductase (GR) activity (Fig. S7), which is necessary to protect cells from oxidative stress [28]. Thus, the abundance of NADP+ markedly increased in the 800 μM-MeP treatment (with FC of 1.448). At the downstream of GSH metabolism, cysteine increased in the 800 μM-MeP treatment, with an FC of 1.387. These results confirm that MeP exposure increased ROS and upregulated GSH metabolism (Fig. 5B).

### 3.2 Cell viability and metabolomics changes induced by hypoxia treatment

Mimicking the intra-mask environment [11], HaCaT cells were cultured under hypoxia for 48 h. No significant change in cell viability was observed in the 48-h hypoxia-treated cells compared to the control (Fig. S8). However, hypoxia led to the generation of ROS (Fig. S3), indicating a disturbance in the equilibrium between oxidants and antioxidants. Mitochondria augment the release of ROS signals to the cytosol during hypoxia, activating diverse protective systems to adapt [29].

**Fig. 2:**
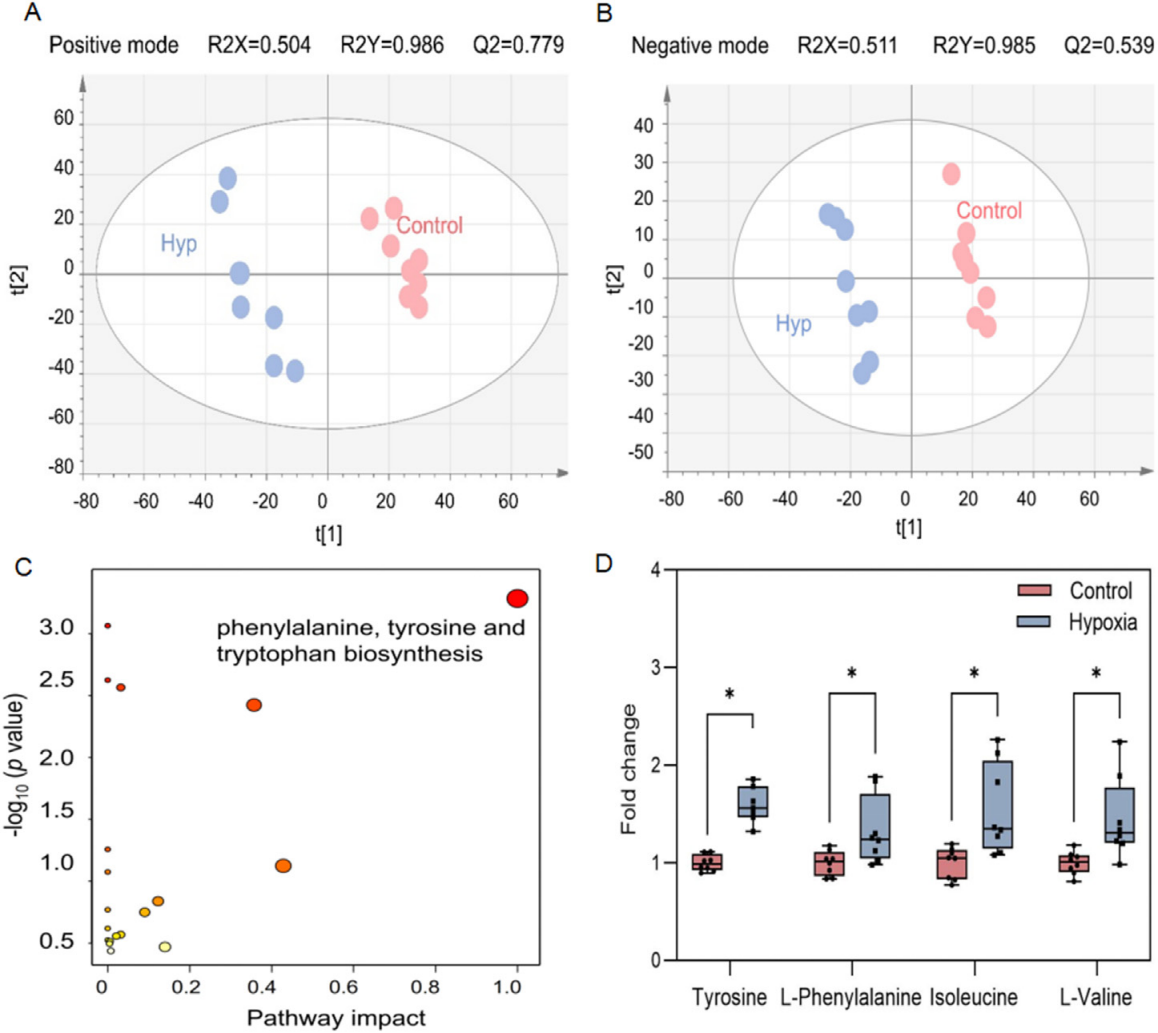
Three-dimensional PLS-DA score plots of HaCaT extract in the control and hyp groups in positive (A) and negative (B) modes. R2Y and Q2 values of the two established models were >0.5, suggesting the good fit and predictive power of the PLS-DA models. (C) Metabolic pathway enrichment was identified among differentially expressed metabolites in the hyp-treated HaCaT cell. (D) Fold change of related metabolites in phenylalanine, tyrosine and tryptophan biosynthesis (tyrosine, l-phenylalanine), isoleucine, and l-valine, ∗*p* < 0.05.

After 48 h hypoxia treatment, a clear separation between the control and hyp group was shown using PLS-DA mode (Fig. 2A and B). After MS^2^ spectra matching, a total of 17 potential biomarkers related to cell hypoxia at the super-class level were identified (Tables S3 and S4), including nucleosides, nucleotides, and analogues (6/17), organic acids and derivatives (6/17), lipids and lipid-like molecules (2/17), organoheterocyclic compounds (2/17), and organic nitrogen compound (1/17).

According to the pathway analysis, phenylalanine, tyrosine, and tryptophan biosynthesis pathways were highly associated with hypoxia treatment in HaCaT cells (Fig. 2C). Tyrosine and l-phenylalanine were significantly increased, with FCs of 1.381 and 1.267 in the hyp group, respectively (Fig. 2D and Table S4). The abundance of tyrosine increased, which might be associated with regulating oxidative stress and inflammation to protect against damage [30,31]. l-phenylalanine is the precursor for tyrosine, which was increased as it needed to be converted to tyrosine [30]. Furthermore, isoleucine and l-valine, two branched-chain amino acids, increased after hypoxic treatment with FCs of 1.447 and 1.380 (Fig. 2C and Table S4), respectively. The branched-chain amino acids are essential to energy metabolism, and the increase of branched-chain amino acids could compensate for the reduction in energy due to hypoxia [30]. Changes in amino acids reflected metabolic remodeling to fulfill the energy requirements after hypoxia treatment.

### 3.3 The combined effect of MeP and hypoxia on cell viability and metabolomics

When the HaCaT cells were jointly treated with hypoxia and MeP (500 μM or 800 μM), the down-regulation of cell viability was not significantly different from that caused by MeP exposure alone (Fig. S8). However, hyp + MeP treatment induced more ROS than single MeP exposure, indicating that hypoxia exacerbated the imbalance of MeP-induced ROS generation (Fig. S3). The co-treatment with hypoxia and MeP led to a more pronounced elevation in SOD activity (Fig. S4A) and a reduction in CAT activity (Fig. S4B). The MDA level in the hyp + 800 μM-MeP treatment was significantly higher than the sum of that in the single 800 μM-MeP and hypoxia groups (Fig. S4C), which is also evidence that hypoxia exacerbated the lipid peroxidation in normal keratinocytes caused by MeP exposure, suggesting a synergistic effect.

**Fig. 3:**
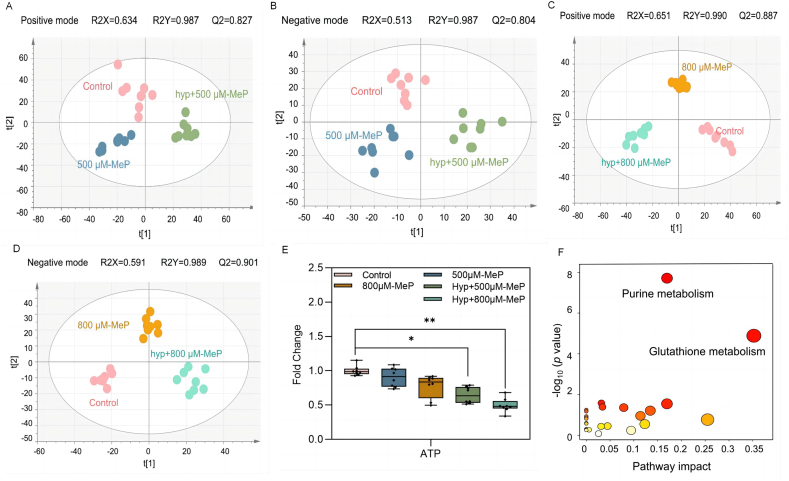
Three-dimensional PLS-DA score plots of HaCaT extract in the control treatment, the 500 μM-MeP treatment, and hyp + 500 μM-MeP treatment in positive (A) and negative (B) modes. Three-dimensional PLS-DA score plots of HaCaT extract in the control treatment, the 800 μM-MeP treatment, and hyp + 800 μM-MeP treatment in positive (C) and negative (D) modes. (E) Fold change of ATP in HaCaT cells after different MeP concentrations and/or hypoxia treatment, ∗∗∗*p* < 0.001. (F) Metabolic pathway analysis of the most relevant metabolite sets in HaCaT cell under the hyp + 800 μM-MeP treatment.

The metabolites distribution of hyp + MeP treatment was distinct from that of MeP treatment and the control, suggesting that hypoxic treatment altered the perturbations of the HaCaT metabolome caused by MeP (R2Y > 0.5, Q2 > 0.5) (Fig. 3A–D). The 40 differential metabolites (*p* < 0.0; FC values > 1.2 or < 0.8) were selected after MeP and hypoxia co-treatment (Tables S3 and S4). These compounds were nucleosides, nucleotides, and analogues (11/40), organic acids and derivatives (10/40), lipids and lipid-like molecules (9/40), organoheterocyclic compounds (5/40), organic nitrogen compounds (3/40), organic oxygen compounds (1/40), and benzenoids (1/40).

Adenosine triphosphate (ATP) is a sensitive marker of cell viability because cells cannot synthesize ATP when they lose membrane integrity and quickly deplete any remaining ATP in the cytoplasm [32]. ATP levels were lower in the hyp + 800 μM-MeP treatment than in the 800 μM-MeP treatment with an FC of 0.352 (Fig. 3E and Table S4), which was consistent with the lower cell viability in the hyp + 800 μM-MeP treatment (Fig. S8). Hypoxia and MeP interacted to influence ATP levels (Table S5). According to pathway analysis, the GSH pathway perturbed by MeP exposure alone was also perturbed in the hyp + 800 μM-MeP treatment (Fig. 3F). However, no significant difference in GSH level was found between the hyp + 800 μM-MeP treated cells and 800 μM-MeP treated cells (Fig. 1D). Perturbations in purine metabolism became more pronounced due to co-treatment with MeP and hypoxic conditions (Fig. 3F). Hypoxanthine is the product of the purine catabolism pathway [33]. Comparing the hyp + 800 μM-MeP co-exposure group with the 800 μM-MeP group, the abundance of hypoxanthine was elevated, with an FC of 3.193 (Table S2). The two-way analysis of variance revealed that hypoxia and MeP interactively influence hypoxanthine (Table S5). The increased level of hypoxanthine might induce ROS generation [34], which requires XOD as an essential enzyme to catalyze hypoxanthine to produce hydrogen peroxide and superoxide anion (oxidation product). The activity of XOD in HaCaT cells was significantly increased after hyp + MeP treatment (Fig. S7), suggesting the possibility of ROS generation. Indeed, ROS production was considerably higher under MeP and hypoxia co-treatment than under MeP treatment alone (Fig. S3). Furthermore, the downstream metabolites of purine metabolism (adenine, ADP, GDP, guanine, guanosine, and xanthosine) increased (Fig. 5B), indicating the activation of purine metabolism and the generation of ROS [35]. The co-treatment of hypoxia and MeP resulted in an obvious disruption in the purine metabolism, which may be the cause of the more pronounced lipid peroxidation.

### 3.4 Inflammation risk in keratinocytes caused by MeP and hypoxia

**Fig. 4:**
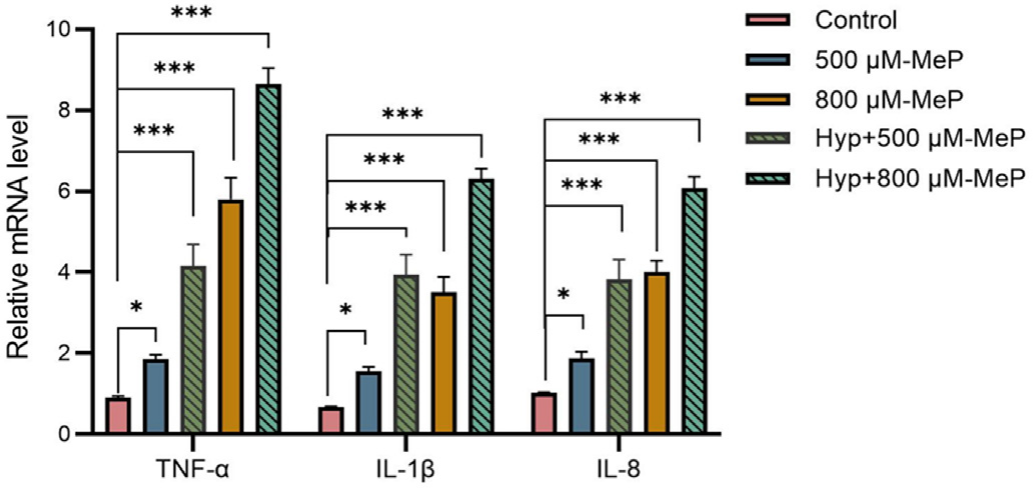
MeP and hypoxia-induced changes of the inflammatory cytokines in HaCaT cells. ∗*p* < 0.05, ∗∗*p* < 0.01, ∗∗∗*p* < 0.001.

Skin is a major producer of cytokines [36]. Oxidative stress can initiate the inflammatory process by activating multiple transcription factors [37]. MeP exposure and hypoxia treatment resulted in the secretion of proinflammatory cytokines, including IL-1β, IL-8, and TNF-α. All the proinflammatory cytokines secreted from HaCaT cells were significantly elevated in the 800 μM-MeP treatment (Fig. 4), especially for the cytokine TNF-α with a 6-fold increase, which suggests that exposure to parabens can stimulate inflammation in keratinocytes, consistent with the observation in autism rat models [38]. Hypoxia has been shown to release TNF-α [39]. The combined treatment of hyp + MeP showed synergistic toxic effects at 500 μM and 800 μM of MeP (Fig. 4). The expression level of TNF-α and IL-1β in the hyp + 800 μM-MeP treatment increased nearly 2-fold compared to the 800 μM-MeP group. TNF-α is a multifunctional proinflammatory factor that induces lymphocytes to release many cytokines and significantly promotes the release of thymic stromal lymphopoietin, disrupting epidermal morphology and barrier function [40]. IL-1β is a cytokine keratinocytes released after epidermal barrier disruption [41]. MeP exposure caused substantial oxidative stress, and hypoxia exacerbated ROS production and might inhibit cellular self-repair [42], which increased proinflammatory cytokines and disrupted the skin barrier.

**Fig. 5:**
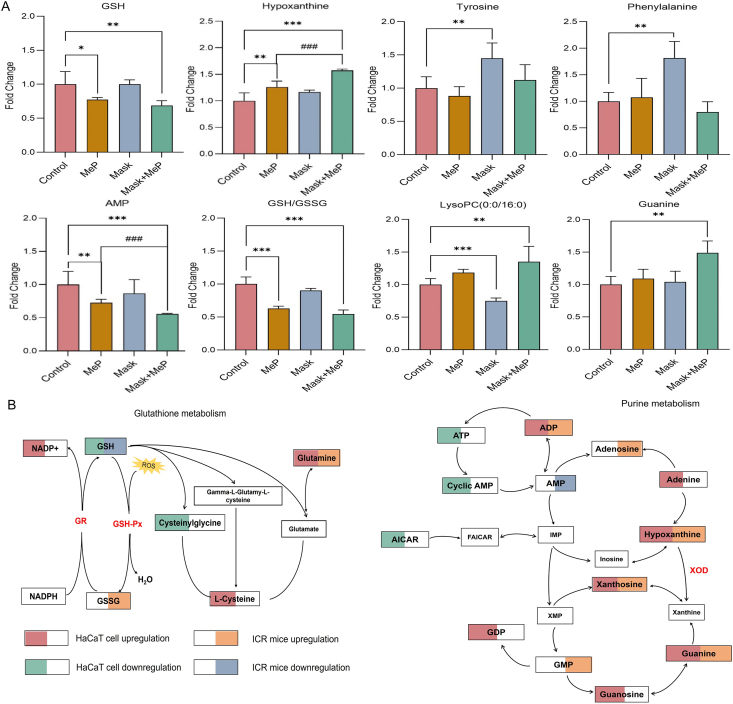
(A) Fold change of representative metabolite changes in the skin tissues of ICR mice exposed to MeP, mask, and mask + MeP. ∗*p* < 0.05, ∗∗*p* < 0.01, ∗∗∗*p* < 0.001, ^###^*p* < 0.001. (B) Changes in related metabolites in GSH metabolism and purine metabolism pathways in HaCaT cell and ICR mice models of the MeP and hyp(mask) + MeP treatment.

### 3.5 Skin metabolic disorder in ICR mice induced by MeP exposure and “mask-wearing”

The skin metabolomic changes caused by mask and MeP exposure were further confirmed in mice models. Untargeted metabolomics analysis showed good separation among MeP treatment, mask treatment, mask + MeP treatment, and the control (Fig. S9), indicating that MeP and mask-wearing conspicuously perturbed endogenous metabolite profiles in mice skin. When exposed to MeP alone, seven metabolites of mice skin were impaired, including representative purine metabolites (hypoxanthine and adenosine monophosphate) and representative GSH metabolites (guanine, GSH, and GSSG). Hypoxanthine was elevated with an FC of 1.26 in the skin of the MeP-exposed mice compared to the control, whereas AMP levels were decreased (FC 0.73) (Fig. 5A). Decreases of GSH (FC 0.77) and GSH/GSSG (FC 0.63) were also found in the skin of mice exposed to MeP (Fig. 5A). Overall, purine and GSH metabolisms were upregulated in the skin of MeP-exposed mice, indicating oxidative stress in the mice's skin, consistent with the results observed in the HaCaT cell (Fig. 5B).

The closed environment within the mask might interfere with human skin metabolomics by disturbing glycerophospholipid and sphingolipid metabolism [12]. When the mice's skin was covered with a mask for 48 h, two metabolites [LysoPC (0:0/16:0), palmitoylcarnitine] in the mice's skin were downregulated, and three metabolites (cytosine, phenylalanine, tyrosine) were upregulated. LysoPC plays a crucial role in maintaining the skin barrier function. The level of LysoPC (0:0/16:0) was reduced with an FC of 0.75 in the skin of mice wearing masks, corroborating our findings observed in the mask-wearing volunteers [12]. The levels of phenylalanine (FC 1.81) and tyrosine (FC 1.45) in the skin of mice “wearing” masks were significantly elevated (Fig. 5A), which was consistent with the results of cellular hypoxic environmental treatment (Fig. 2D).

The perturbation of metabolites in the mice's skin related to MeP exposure was more obvious under the hypoxic environment created by the mask “wearing”. Notably, compared to the alone MeP treatment, the downregulation of AMP (FC 0.77) and the upregulation of hypoxanthine (FC 1.25) were more pronounced (Fig. 5A). Furthermore, guanine, which is downstream in purine metabolism, was significantly upregulated compared with that of the MeP treatment (Fig. 5A). A hermetic hypoxic environment created by the mask could exacerbate the purine metabolic interference of MeP on the skin of mice, which was consistent with the *in vitro* results obtained from skin HaCaT cell cultures (Fig. 5B).

Based on the results of HaCaT cell and ICR mice models, MeP exposure leads to a significant upregulation of GSH metabolism in the skin. The hypoxic environment formed by wearing masks may result in the upregulation of amino acids related to the phenylalanine, tyrosine, and tryptophan biosynthesis pathways of the skin. In addition, the hypoxic environment could increase the effect of MeP on the upregulation of purine metabolism, demonstrating a synergistic effect on skin health.

The alterations in the skin metabolomics also indicated the presence of oxidative stress in the mice's skin. Mask + MeP treatment induced more ROS production than single MeP exposure, indicating hypoxia caused by “mask-wearing” exacerbated the imbalance of MeP-induced ROS generation (Fig. S10). Additionally, the MDA level in the mask + MeP treatment was significantly higher than the control group (Fig. S11), which was also consistent with the results of cellular treatment (Fig. S4C).

### 3.6 Network toxicology analysis of the target genes related to MeP and hypoxia in skin

**Fig. 6:**
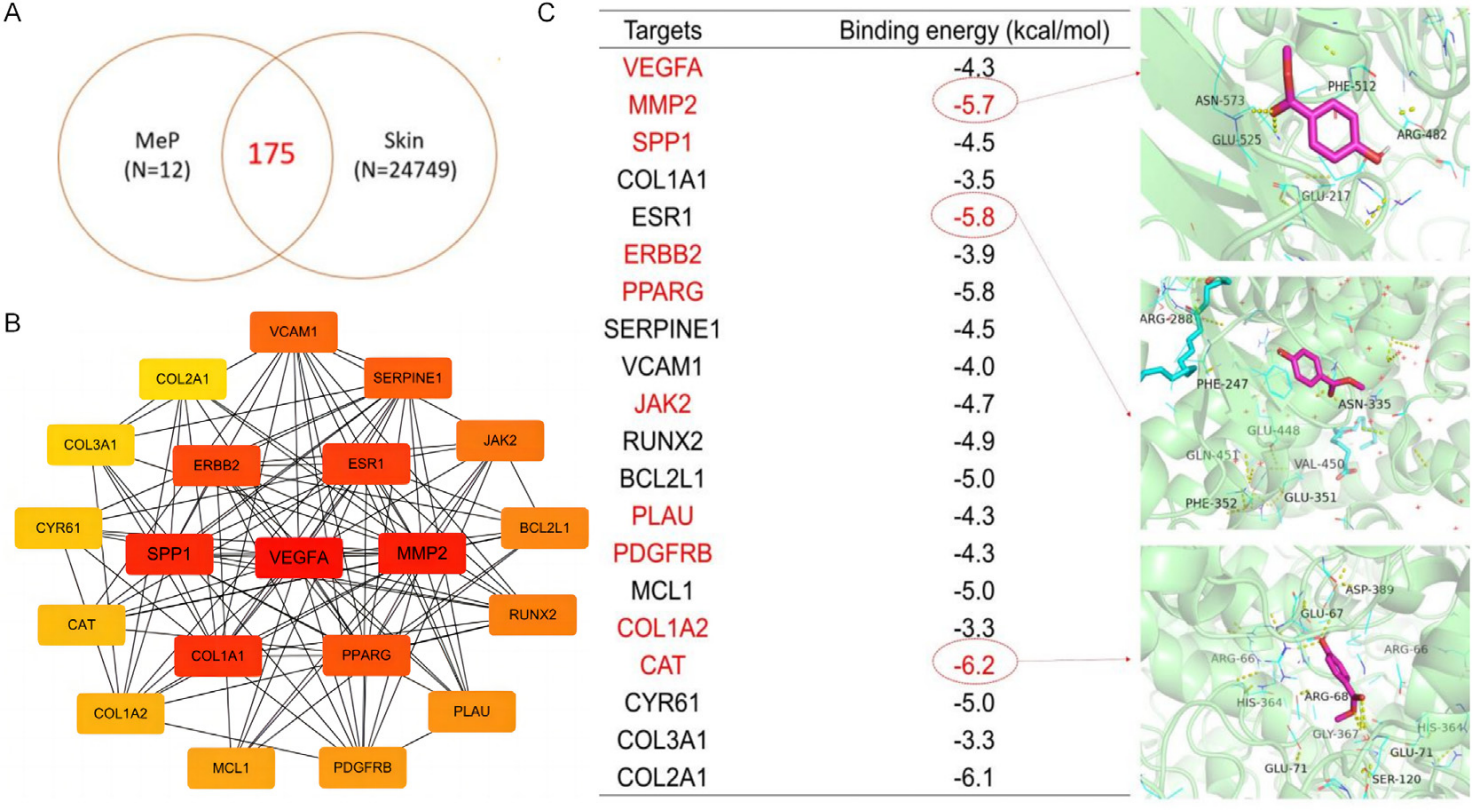
(A) Venn diagram for screening of “common targets”. (B) PPI network of top 20 candidate gene targets for skin health risk induced by MeP. The redder the nodes indicate that genes are more critical in the interaction. (C) The virtual docking of MeP with top 10 candidate targets, and the docking conformation of the MeP and CAT, MeP and PPARG, MeP and MMP2.

A total of 187 chemical target genes were identified by screening MeP targets using the comparative toxicology genomics database and the Swiss target prediction database, among which 175 genes are health risk target genes related to the skin identified by GeneCards and OMIM database. Therefore, these 175 genes were considered candidate target genes for skin health risks caused by MeP (Fig. 6A). Data were put into Cytoscape 3.9.1 to draw the “MeP-targets-risk” network (Fig. S12). According to cytoHubba analysis, VEGFA, MMP2, SPP1, COL1A1, ESR1, ERBB2, PPARG, SERPINE1, VCAM1, JAK2, RUNX2, BCL2L1, PLAU, PDGFRB, MCL1, COL1A2, CAT, CYR61, COL3A1 and COL2A1 may be the top 20 candidate targets which more related to MeP (Fig. 6B), with the score shown in Table S6.

Combining the 20 candidate target genes and the 40 differential metabolites induced by MeP exposure by a gene–metabolite interaction network analysis, the association between VEGFA, MMP2, SPP1, ERBB2, PPARG, JAK2, PLAU, PDGFRB, COL1A2, and CAT with different metabolites were found (Fig. S13). Molecular docking was applied to evaluate the binding ability of MeP to target genes (Fig. 6C). CAT was the target with the highest binding energy (−6.2 kcal/mol). As an antioxidant, the CAT gene codes for creating the enzymes necessary to neutralize H_2_O_2_. The screening of the CAT target gene was consistent with the result of increased ROS induced by MeP exposure (Figs. S3 and S4B). PPARG and MMP2 were the second and third highest binding activity target genes with MeP, with a binding energy of −5.8 and −5.7 kcal/mol, respectively (Fig. 6C). PPARG is one of the transcription factors regulating keratinocyte differentiation, while MMP2 is required for human keratinocyte proliferation [43]. All these three major target genes might be involved in the biological process of cellular response to MeP stress.

The CAT gene expression was likely activated at the hypoxic stress level to maintain cellular homeostasis [44]. MMP2 and PPARG were also significantly downregulated and induced by hypoxia [45]. CAT [46] and MMP2 [47] are associated with skin aging, while PPARG is associated with skin barrier damage [48]. MeP and hypoxia co-regulated shared genes and might further increase skin health risks.

## 4 Conclusions

Overall, excessive exposure to MeP, which is commonly added to personal care products, could disturb skin GSH metabolism to respond to oxidative stress. The hypoxic environment could increase the effect of MeP on the upregulation of purine metabolism and exacerbate the production of ROS, thereby causing more significant lipid peroxidation and skin inflammation, indicative of a synergistic effect on skin health. Network toxicology analysis identified CAT, PPARG, and MMP2 genes as possible key gene targets responsible for this synergistic effect. The results helped improve our understanding of the mechanism of mask-wearing and preservative-related dermal toxicity at a molecular level.

## Author contributions

Y.L.: conceptualization, investigation, methodology, formal analysis, visualization, writing–original draft. L.J.C.: methodology, formal analysis, visualization, writing–review & editing. S.Y.Z. and X.X.W: methodology. Y.Y.S: formal analysis. H.W.S.: conceptualization. Z.W.C.: conceptualization, supervision. L.W.: conceptualization, supervision, funding acquisition, writing–review & editing.

## Declaration of competing interests

The authors declare no conflict of interests.

## Acknowledgments

This work was supported by the 10.13039/501100001809National Natural Science Foundation of China (U22A20614 & 41722304) and the Ministry of Education, China (T2017002).

## References

[1] Dabrowska A.K., Spano F., Derler S., Adlhart C., Spencer N.D. (2018). The relationship between skin function, barrier properties, and body-dependent factors. Skin Res. Technol..

[2] Petric Z., Ruzic J., Zuntar I. (2021). The controversies of parabens - an overview nowadays. Acta Pharm..

[3] Van der Schyff V., Suchankova L., Kademoglou K., Melymuk L., Klanova J. (2022). Parabens and antimicrobial compounds in conventional and “green” personal care products. Chemosphere.

[4] Pažoureková S., Hojerová J., Klimová Z., Lucová M. (2013). Dermal absorption and hydrolysis of methylparaben in different vehicles through intact and damaged skin: using a pig-ear model in vitro. Food Chem. Toxicol..

[5] Nishizawa C., Takeshita K., Ueda J., Nakanishi I., Suzuki K.T. (2006). Reaction of para-hydroxybenzoic acid esters with singlet oxygen in the presence of glutathione produces glutathione conjugates of hydroquinone, potent inducers of oxidative stress. Free Radic. Res..

[6] Handa O., Kokura S., Adachi S., Takagi T., Naito Y. (2006). Methylparaben potentiates UV-induced damage of skin keratinocytes. Toxicology.

[7] Stucker M., Struk A., Altmeyer P., Herde M., Baumgartl H. (2002). The cutaneous uptake of atmospheric oxygen contributes significantly to the oxygen supply of human dermis and epidermis. J. Physiol..

[8] Golja P. (2004).

[9] Bedogni B., Powell M.B. (2006). Skin hypoxia: a promoting environmental factor in melanomagenesis. Cell Cycle.

[10] Machcinska S., Walendzik K., Kopcewicz M., Wisniewska J., Rokka A. (2022). Hypoxia reveals a new function of Foxn1 in the keratinocyte antioxidant defense system. FASEB J..

[11] Maura I.Y., Zabala D.D., Cuixart D.B., Rincon J.A.G., Turcó J.V. (2022). The physiological impact of different types of mask at rest. Apunts Sports Med..

[12] Liu Y., Zhao H.Z., Chen H., Li X.X., Ran C.M. (2023). Does mask wearing affect skin health? An untargeted skin metabolomics study. Environ. Int..

[13] Mieremet A., Garcia A.V., Boiten W., van Dijk R., Gooris G. (2019). Human skin equivalents cultured under hypoxia display enhanced epidermal morphogenesis and lipid barrier formation. Sci. Rep..

[14] Nys K., Maes H., Andrei G., Snoeck R., Garmyn M. (2012). Skin mild hypoxia enhances killing of UVB-damaged keratinocytes through reactive oxygen species-mediated apoptosis requiring Noxa and Bim. Free Radic. Biol. Med..

[15] Tesfaldet Y.T., Ndeh N.T. (2022). Public face masks wearing during the COVID-19 pandemic: a comprehensive analysis is needed for potential implications. J. Hazard. Mater. Adv..

[16] Korrapati N.H., Perera M.H., Swamy P.K., Ranganath P.A., Ankireddy K. (2021). Skin-care routine during the COVID-19 pandemic: an online survey. Int. J. Progress. Sci. Technol..

[17] Mundy R.D., Cormack B. (2009). Expression of Candida glabrata adhesins after exposure to chemical preservatives. J. Infect. Dis..

[18] Ramirez T., Daneshian M., Kamp H., Bois F.Y., Clench M.R. (2013). Metabolomics in toxicology and preclinical research. ALTEX.

[19] Randhawa M., Southall M., Samaras S.T. (2013). Metabolomic analysis of sun exposed skin. Mol. Biosyst..

[20] Zhao L., Zhang H., Li N., Chen J., Xu H. (2013). Network pharmacology, a promising approach to reveal the pharmacology mechanism of Chinese medicine formula. J. Ethnopharmacol..

[21] Li X.Y., Jin X., Li Y.Z., Gao D.D., Liu R. (2019). Network toxicology and LC-MS-based metabolomics: new approaches for mechanism of action of toxic components in traditional Chinese medicines. Chin. Herb. Med..

[22] Zheng Y.C., Li X.K., Yan R., Deng S., Li M.Y. (2021). Evaluation of biological mechanisms of eucommiae folium in hypertensive kidney injury by integration of untargeted metabolomics and network pharmacology. J. Proteome Res..

[23] Luan H.M., Liu L.F., Meng N., Tang Z., Chua K.K. (2015). LC MS-based urinary metabolite signatures in idiopathic Parkinson's disease. J. Proteome Res..

[24] Liang Y.S., Zhang H.N., Cai Z.W. (2021). New insights into the cellular mechanism of triclosan-induced dermal toxicity from a combined metabolomic and lipidomic approach. Sci. Total Environ..

[25] Lin W.Y., Wang C.Q., Liu G.P., Bi C., Wang X. (2020). SLC7A11/xCT in cancer: biological functions and therapeutic implications. Am. J. Cancer Res..

[26] Kizhedath A., Wilkinson S., Glassey J. (2019). Assessment of hepatotoxicity and dermal toxicity of butylparaben and methyl paraben using HepG2 and HDFn in vitro models. Toxicol. In Vitro.

[27] Jones D.P. (2006). Extracellular redox state: refining the definition of oxidative stress in aging. Rejuvenation Res..

[28] Silva D.C., Serrano L., Oliveira T.M.A., Mansano A.S., Almeida E.A. (2018). Effects of parabens on antioxidant system and oxidative damages in nile tilapia (Oreochromis niloticus). Ecotoxicol. Environ. Saf..

[29] Smith K.A., Waypa G.B., Schumacker P.T. (2017). Redox signaling during hypoxia in mammalian cells. Redox Biol..

[30] Gandhi S., Chinnadurai V., Bhadra K., Gupta I., Kanwar R.S. (2022). Urinary metabolic modulation in human participants residing in Siachen: a 1H NMR metabolomics approach. Sci. Rep..

[31] Chicco A.J., Le C.H., Gnaiger E., Dreyer H.C., Muyskens J.B. (2018). Adaptive remodeling of skeletal muscle energy metabolism in high-altitude hypoxia: lessons from AltitudeOmics. J. Biol. Chem..

[32] Riss T.L., Moravec R.A., Niles A.L. (2011). Cytotoxicity testing: measuring viable cells, dead cells, and detecting mechanism of cell death. Methods Mol. Biol..

[33] Fox I.H. (1988). Metabolic basis for disorders of purine nucleotide degradation. Metabolism.

[34] Ryu H.M., Kim Y.J., Oh E.J., Oh S.H., Choi J.Y. (2016). Hypoxanthine induces cholesterol accumulation and incites atherosclerosis in apolipoprotein E-deficient mice and cells. J. Cell. Mol. Med..

[35] Agarwal A., Banerjee A., Banerjee U.C. (2011). Xanthine oxidoreductase: a journey from purine metabolism to cardiovascular excitation contraction coupling. Crit. Rev. Biotechnol..

[36] Gröne A. (2002). Keratinocytes and cytokines. Vet. Immunol. Immunopathol..

[37] Zhong Z.Y., Zhai Y.G., Liang S., Mori Y.S., Han R.Z. (2013). TRPM2 links oxidative stress to NLRP3 inflammasome activation. Nat. Commun..

[38] Hegazy H.G., Ali E.H.A., Elgoly A.H.M. (2015). Interplay between proinflammatory cytokines and brain oxidative stress biomarkers: evidence of parallels between butyl paraben intoxication and the valproic acid brain physiopathology in autism rat model. Cytokine.

[39] Smith R.M., McCarthy J., Sack M. (2001). TNF alpha is required for hypoxia-mediated right ventricular hypertrophy. Mol. Cell. Biochem..

[40] Kim J., Kim B.E., Leung D.Y.M. (2019). Pathophysiology of atopic dermatitis: clinical implications. Allergy Asthma Proc..

[41] Barker J.N., Jones M.L., Mitra R.S., Crockett-Torabe E., Fantone J.C. (1991). Modulation of keratinocyte-derived interleukin-8 which is chemotactic for neutrophils and T lymphocytes. Am. J. Pathol..

[42] Buravkova L.B., Andreeva E., Gogvadze V., Zhivotovsky B. (2014). Mesenchymal stem cells and hypoxia: where are we?. Mitochondrion.

[43] Xue M.L., Jackson C.J. (2008). Autocrine actions of matrix metalloproteinase (MMP)-2 counter the effects of MMP-9 to promote survival and prevent terminal differentiation of cultured human keratinocytes. J. Invest. Dermatol..

[44] Gostyukhina O.L., Yu A.A., Chelebieva E.S., Vodiasova E.A., Lantushenko A.O. (2022). Adaptive potential of the mediterranean mussel mytilus galloprovincialis to short-term environmental hypoxia. Fish Shellfish Immunol..

[45] Anvari G., Bellas E. (2021). Hypoxia induces stress fiber formation in adipocytes in the early stage of obesity. Sci. Rep..

[46] Pluemsamran T., Onkoksoong T., Panich U. (2012). Caffeic acid and ferulic acid inhibit UVA-induced matrix metalloproteinase-1 through regulation of antioxidant defense system in keratinocyte HaCaT cells. Photochem. Photobiol..

[47] Lan Y., Wang Y., Lu H. (2020). Opsin 3 is a key regulator of ultraviolet A-induced photoageing in human dermal fibroblast cells. Br. J. Dermatol..

[48] Blunder S., Moosbrugger-Martinz V., Gruber R., Schmuth M., Dubrac S. (2016). Skin barrier impairment downregulates PPARG via IL-1β. J. Invest. Dermatol..

