# Supplementary material for: Do wearing masks and preservatives have a combined effect on skin health?
Source: Eco Environ Health. 2024 Jan 24;3(1):107–15. doi: 10.1016/j.eehl.2024.01.003 (PMC10912353; doi:10.1016/j.eehl.2024.01.003)
Supplement: Multimedia component 1 [file mmc1.docx]

**SUPPORTING INFORMATION**

**Do wearing mask and preservative have a combined effect on skin health?**

Yu Liu ^†,‡,1^, Leijian Chen ^‡,1^, Shuyi Zhang ^†^, Xiaoxiao Wang ^‡^, Yuanyuan Song ^‡^, Hongwen Sun ^†^, Zongwei Cai ^‡,*^, Lei Wang ^†,*^

† MOE Key Laboratory of Pollution Processes and Environmental Criteria, College of Environmental Science and Engineering, Nankai University, Tianjin 300350, China

‡ State Key Laboratory of Environmental and Biological Analysis, Department of Chemistry, Hong Kong Baptist University, Hong Kong 999077, China

^1^ These authors contribute equally to this work.

*Number of pages: 23*

*Number of tables: 6*

*Number of figures: 13*

**Table S1 PCR primers and amplification condition~~s~~**

| mRNA | Primer sequence | | PCR condition |
| --- | --- | --- | --- |
|  | upstream sequence | downstream sequence |  |
| GAPDH | GTCAGTGGTGGACCTGACCT | AGGGGTCTACATGGCAACTG | Pre-denaturation: 95 ℃, 2 minutes  Denaturation: 95 ℃, 5s, 40 cycles  Extension: 60 ℃, 30–34s, 40 cycles  Annealing: 60 ℃, 30–34s, 40 cycles |
| TNF-α | CTGGGCAGGTCTACTTTGGG | TCCCAGGTTTCGAAGTGGTG |  |
| 1L-1β | AAAAGCTTGGTGATGTCTGG | TTTCAACACGCAGGACAGG |  |
| 1L-8 | ATGATCTCCAAGCTGGGCCGTG | TATGAATTCTCAGCCCTCTTCAAAA |  |

**Table S2 Instrumental method for the analysis of untargeted metabolomics samples by UHPLC-Orbitrap-HRMS**

| Contents | Parameters |
| --- | --- |
| Mobile Phases | A. water + 0.1% FA  B. ACN + 0.1% FA |
| Gradient Profile | 0–1 min: 2% B;  1-19 min: 2% B–100% B;  19–21 min: 100% B;  21–21.1 min: 100% B–22% B;  21.1–25 min: 2% B. |
| Flow rate | 0.30 mL/min |
| MS Parameters | Full scan mode  Resolution = 50000  Scan range (m/z) = 70–1000  Spray voltage (kV) = 3.5 (positive ionization mode)  2.6 (negative ionization mode)  Sheath gas flow rate (arbitrary units) = 40  Auxiliary gas flow rate (arbitrary units) = 10  Auxiliary gas heater temperature (°C) = 320  Capillary temperature (°C) = 320 |
| MS/MS Parameters | Higher-energy collisional dissociation (HCD) mode  Collision energy (eV) = 10, 25, 40 |

**Table S3 The identification information of differential metabolites**

| Compounds | RT (min) | *m/z* | MS pattern | HMDB NO. |
| --- | --- | --- | --- | --- |
| Adenine | 3.64 | 136.0620 (+) | 92.0242, 94.0399, 119.0356 | HMDB0000034 |
| Adenosine | 3.66 | 268.1045 (+) | 136.0621 | HMDB0000050 |
| ADP | 1.70 | 428.0371 (+) | 97.0287, 136.0620, 348.0705 | HMDB0001341 |
| AICAR | 11.70 | 337.0530 (-) | 337.0530 | HMDB0001517 |
| ATP | 1.66 | 508.0039 (+) | 97.0288, 136.0620, 330.0602, 410.0269 | HMDB0000538 |
| Azelaic acid | 8.38 | 187.0965 (-) | 57.0337, 97.0653, 125.0965 | HMDB0000784 |
| Citric acid | 2.18 | 191.0188 (-) | 67.0184, 87.0082, 111.0080, 129.0186 | HMDB0000094 |
| Corticosterone | 15.12 | 347.2206 (+) | 347.2206 | HMDB0001547 |
| Creatinine | 1.20 | 114.0665 (+) | 72.0445, 86.0717 | HMDB0000562 |
| Cyclic AMP | 3.88 | 330.0601 (+) | 136.0618 | HMDB0000058 |
| Cysteinylglycine | 1.91 | 179.0483 (+) | 58.9953, 76.0218, 116.0167, 144.0114, 162.0221 | HMDB0000078 |
| CMP | 1.19 | 324.0601 (+) | 97.0285, 112.0509 | HMDB0000095 |
| Decanoylcarnitine | 11.11 | 316.2487 (+) | 85.0288, 257.1748 | HMDB0000651 |
| Docosahexaenoic acid | 17.95 | 341.2667 (+) | 341.2667 | HMDB0002183 |
| gamma-Glutamylglutamic acid | 1.25 | 277.1068 (+) | 84.0448, 130.0499, 148.0608, 214.0708 | HMDB0011737 |
| GDP | 1.73 | 444.0329 (+) | 97.0287, 152.0570 | HMDB0001201 |
| Glutathione | 1.86 | 308.2917 (+) | 76.0219, 162.0223, 233.0594 | HMDB0062697 |
| Glutamine | 1.13 | 147.0768 (+) | 56.0498, 84.0448, 130.0502 | HMDB0000641 |
| Guanine | 3.84 | 152.0570 (+) | 82.0402, 110.0353 | HMDB0000132 |
| Guanosine | 3.92 | 282.0833 (-) | 107.0353, 108.0197, 133.0148, 150.0414 | HMDB0000133 |
| 13-HODE | 15.67 | 295.2278 (-) | 295.2278 | HMDB0004667 |
| Hypoxanthine | 3.86 | 137.0461 (+) | 67.0291, 82.0400, 94.0403, 110.0352 | HMDB0000157 |
| Isoleucine | 2.49 | 132.1021 (+) | 58.0652, 69.0702, 86.0968 | HMDB0000172 |
| L-Acetylcarnitine | 1.78 | 204.1233 (+) | 60.0811, 85.0287, 145.0498 | HMDB0000201 |
| L-Carnitine | 1.16 | 162.1127 (+) | 60.0811, 85.0288, 103.0393 | HMDB0000062 |
| L-Cysteine | 1.18 | 122.0276 (+) | 58.9953, 76.0219, 105.0004 | HMDB0000574 |
| L-Kynurenine | 4.28 | 209.0928 (+) | 94.0654, 120.0446, 146.0604, 174.0552 | HMDB0000684 |
| L-Phenylalanine | 4.33 | 166.0866 (+) | 79.0546, 93.0702, 103.0546, 120.0811, 131.0495, 149.0595 | HMDB0000159 |
| L-Valine | 1.74 | 118.0866 (+) | 55.0546, 57.0576, 72.0811 | HMDB0000883 |
| Malic acid | 1.34 | 133.0143 (-) | 71.0133, 89.0238, 115.0029 | HMDB0000156 |
| 3-Methylpentanoylcarnitine/4-Methylpentanoylcarnitine | 7.43 | 260.1859 (+) | 85.0287, 99.0805, 144.1018, 201.1121 | HMDB0241033/HMDB0241032 |
| 5'-Methylthioadenosine | 5.17 | 298.0966 (+) | 61.0108, 97.0287, 136.0621, 163.0422 | HMDB0001173 |
| NADP+ | 1.72 | 744.0847 (+) | 136.0620, 312.0481 | HMDB0000217 |
| Oleamide | 19.19 | 282.2796 (+) | 83.0859, 121.1014, 247.2420 | HMDB0002117 |
| Oxidized glutathione | 1.22 | 613.1599 (+) | 177.0328, 235.0210, 484.1181 | HMDB0003337 |
| Palmitoylcarnitine | 15.39 | 400.3425 (+) | 85.0287, 341.2694 | HMDB0000222 |
| Phosphocreatine | 1.27 | 210.0278 (-) | 78.9584 | HMDB0001511 |
| S-Adenosylmethionine | 1.15 | 399.1456 (+) | 97.0287, 136.0623, 250.0935, 298.0981 | HMDB0001185 |
| Spermidine | 0.95 | 146.1655 (+) | 72.0811, 75.0920, 112.1124, 129.1386 | HMDB0001257 |
| Sphingosine | 13.69 | 282.2795 (+) | 83.0855, 252.2694 | HMDB0000252 |
| Taurine | 1.14 | 126.0222 (+) | 108.0117 | HMDB0000251 |
| Tyrosine | 2.85 | 165.0550 (+) | 91.0546, 123.0444, 136.0760, 14 7.0444 | HMDB0000158 |
| Uracil | 2.63 | 113.0348 (+) | 70.0291, 95.0240 | HMDB0000300 |
| UDP -N-acetylglucosamine | 1.54 | 606.0732 (-) | 78.9585, 158.9246, 282.0372, 402.9943 | HMDB0000290 |
| Urocanic acid | 1.84 | 139.0505 (+) | 68.0496, 93.0450, 121.0400 | HMDB0000301 |
| Xanthosine | 4.22 | 283.0672 (-) | 151.0252 | HMDB0000299 |

**Table S4 Statistical analysis of differential metabolites in different exposure treatment**

| metabolites | 500 μM-MeP *vs.* con | |  | 800 μM-MeP *vs.* con | |  | hyp *vs.* con | |  | hyp+500 μM-MeP *vs.* con | |  | hyp+800 μM-Mep *vs.* con | |
| --- | --- | --- | --- | --- | --- | --- | --- | --- | --- | --- | --- | --- | --- | --- |
|  | FC | *P* value |  | FC | *P* value |  | FC | *P* value |  | FC | *P* value |  | FC | *P* value |
| Adenine | 0.997 | 0.753 |  | 0.988 | 0.753 |  | 1.127 | 0.141 |  | 1.271 | 0.115 |  | 1.414 | ** |
| Adenosine | 1.336 | 0.074 |  | 1.311 | 0.141 |  | 1.498 | * |  | 1.374 | 0.074 |  | 1.254 | 0.294 |
| ADP | 0.945 | 0.208 |  | 1.041 | 0.916 |  | 1.533 | * |  | 1.337 | 0.248 |  | 2.077 | ** |
| AICAR | 0.956 | 0.834 |  | 0.928 | 0.674 |  | 0.647 | 0.141 |  | 0.848 | 0.401 |  | 0.546 | * |
| ATP | 0.906 | 0.834 |  | 0.776 | 0.529 |  | 0.591 | 0.052 |  | 0.494 | * |  | 0.373 | ** |
| Azelaic acid | 0.926 | 0.462 |  | 0.069 | ** |  | 0.862 | 1.000 |  | 0.834 | 0.834 |  | 0.071 | ** |
| Citric acid | 1.339 | * |  | 1.193 | * |  | 1.527 | * |  | 1.633 | ** |  | 1.442 | ** |
| Corticosterone | 0.687 | 0.059 |  | 0.714 | 0.059 |  | 0.682 | 0.401 |  | 0.504 | ** |  | 0.306 | ** |
| Creatinine | 0.930 | 0.916 |  | 0.936 | 1.000 |  | 0.613 | 0.294 |  | 0.484 | * |  | 0.459 | ** |
| Cyclic AMP | 0.782 | ** |  | 0.494 | ** |  | 0.695 | ** |  | 0.756 | ** |  | 0.223 | ** |
| Cysteinylglycine | 0.953 | 0.916 |  | 0.647 | ** |  | 0.877 | 0.600 |  | 0.881 | 0.172 |  | 0.699 | ** |
| CMP | 0.937 | 0.208 |  | 0.997 | 0.529 |  | 1.535 | ** |  | 1.512 | * |  | 2.725 | ** |
| Decanoylcarnitine | 0.974 | 0.401 |  | 0.555 | ** |  | 1.362 | 0.600 |  | 0.933 | 0.462 |  | 0.639 | * |
| Docosahexaenoic acid | 0.689 | * |  | 0.549 | ** |  | 0.487 | ** |  | 0.666 | * |  | 0.253 | ** |
| gamma-Glutamylglutamic acid | 0.958 | 0.916 |  | 1.246 | 0.462 |  | 0.897 | 0.600 |  | 1.067 | 0.834 |  | 2.897 | ** |
| GDP | 1.061 | 0.834 |  | 1.191 | 0.294 |  | 1.345 | 0.059 |  | 1.145 | 0.753 |  | 1.735 | ** |
| Glutathione | 0.971 | 0.753 |  | 0.714 | ** |  | 0.886 | 0.462 |  | 0.924 | 0.600 |  | 0.658 | ** |
| Glutamine | 1.348 | * |  | 1.411 | * |  | 0.991 | 0.753 |  | 1.367 | * |  | 1.775 | ** |
| Guanine | 0.742 | 0.916 |  | 0.933 | 0.401 |  | 0.650 | 0.529 |  | 1.020 | 0.248 |  | 2.859 | ** |
| Guanosine | 1.022 | 0.674 |  | 1.644 | 0.059 |  | 1.321 | 0.208 |  | 1.227 | 0.248 |  | 2.816 | ** |
| 13-HODE | 1.092 | 0.093 |  | 0.921 | 0.345 |  | 0.988 | 0.834 |  | 1.032 | 0.753 |  | 1.293 | * |
| Hypoxanthine | 0.984 | 0.529 |  | 1.601 | * |  | 1.049 | 0.600 |  | 1.253 | 0.208 |  | 5.113 | ** |
| Isoleucine | 1.130 | 0.462 |  | 0.961 | 0.401 |  | 1.447 | * |  | 0.880 | 0.345 |  | 0.782 | 0.115 |
| L-Acetylcarnitine | 1.119 | 0.529 |  | 1.523 | * |  | 1.508 | 0.074 |  | 1.518 | * |  | 1.989 | ** |
| L-Carnitine | 1.010 | 0.401 |  | 0.964 | 0.345 |  | 1.707 | * |  | 1.453 | ** |  | 1.503 | ** |
| L-Cysteine | 1.276 | * |  | 1.387 | ** |  | 1.415 | 0.059 |  | 1.610 | * |  | 2.099 | ** |
| L-Kynurenine | 1.398 | * |  | 2.970 | ** |  | 1.311 | 0.345 |  | 2.159 | ** |  | 6.316 | ** |
| L-Phenylalanine | 1.186 | 0.093 |  | 1.141 | 0.248 |  | 1.254 | * |  | 0.985 | 0.916 |  | 1.049 | 0.753 |
| L-Valine | 1.048 | 0.529 |  | 1.362 | 0.059 |  | 1.380 | * |  | 0.927 | 0.529 |  | 0.937 | 0.529 |
| Malic acid | 0.965 | 0.208 |  | 1.033 | 0.834 |  | 1.070 | 0.294 |  | 2.234 | * |  | 4.043 | ** |
| 3-Methylpentanoylcarnitine/4-Methylpentanoylcarnitine | 0.994 | 0.753 |  | 1.148 | 0.294 |  | 1.632 | * |  | 1.673 | ** |  | 1.726 | ** |
| 5'-Methylthioadenosine | 0.549 | ** |  | 0.011 | ** |  | 0.274 | ** |  | 0.092 | ** |  | 0.002 | ** |
| NADP+ | 1.269 | 0.208 |  | 1.448 | * |  | 1.226 | 0.401 |  | 1.182 | 0.345 |  | 1.643 | ** |
| Oleamide | 1.154 | 0.529 |  | 0.902 | 0.401 |  | 1.015 | 0.916 |  | 0.708 | 0.059 |  | 0.369 | ** |
| Oxidized glutathione | 1.028 | 0.345 |  | 0.427 | 0.074 |  | 0.963 | 0.172 |  | 0.978 | 0.600 |  | 0.302 | * |
| Palmitoylcarnitine | 1.169 | 1.000 |  | 0.942 | 0.674 |  | 1.501 | 0.059 |  | 0.987 | 0.916 |  | 0.504 | ** |
| Phosphocreatine | 0.875 | 0.529 |  | 0.592 | ** |  | 0.892 | 1.000 |  | 0.839 | 0.462 |  | 0.590 | ** |
| S-Adenosylmethionine | 1.042 | 0.600 |  | 1.437 | 0.059 |  | 1.363 | 0.074 |  | 1.509 | * |  | 2.014 | * |
| Spermidine | 0.912 | 0.834 |  | 1.045 | 0.115 |  | 0.950 | 0.115 |  | 1.245 | * |  | 1.365 | * |
| Sphingosine | 0.847 | 0.208 |  | 0.773 | 0.172 |  | 0.853 | 0.401 |  | 0.605 | * |  | 0.279 | ** |
| Taurine | 0.987 | 0.834 |  | 1.307 | 0.093 |  | 1.427 | ** |  | 0.884 | 0.600 |  | 0.876 | 0.600 |
| Tyrosine | 1.142 | 0.208 |  | 1.172 | 0.172 |  | 1.381 | * |  | 1.058 | 0.345 |  | 0.998 | 0.600 |
| Uracil | 0.818 | 0.141 |  | 0.963 | 0.834 |  | 1.142 | 0.208 |  | 1.202 | 0.141 |  | 2.911 | ** |
| UDP -N-acetylglucosamine | 1.141 | 0.674 |  | 1.453 | * |  | 1.538 | * |  | 1.653 | ** |  | 1.700 | ** |
| Urocanic acid | 1.025 | 0.916 |  | 3.136 | 0.158 |  | 2.597 | * |  | 2.639 | 0.462 |  | 3.635 | ** |
| Xanthosine | 0.977 | 1.000 |  | 1.691 | 0.059 |  | 1.370 | 0.141 |  | 1.240 | 0.248 |  | 2.930 | ** |

FC: fold change; FC with a value >1.2 represents a relatively higher concentration in MeP (hyp, hyp+MeP) exposure, while FC < 0.8 indicates a relatively lower concentration in MeP (hyp, hyp+MeP) exposure compared with the control group. The significances were calculated with the Mann-Whitney U test, and * and ** represents *P* value of < 0.05 and < 0.01, respectively. Up-regulation and down-regulation were marked in red and green, respectively.

**Table S5** **Two-way analysis of variance to test the effect of MeP and hypoxia on metabolites**

| Target genes name | GSH | ATP | Hypoxanthine |
| --- | --- | --- | --- |
| MeP | *** | ** | *** |
| Hypoxia | 0.075 | *** | *** |
| MeP × Hypoxia | 0.868 | * | ** |

Significant differences were analyzed with a two-way analysis of variance, and *P* values were calculated. *, ** and *** represents *P* value of < 0.05, < 0.01 and < 0.001, respectively

**Table S6 The score of the top 20 candidate target genes**

**according to cytoHubba analysis**

| Target genes name | Score |
| --- | --- |
| VEGFA | 6124 |
| MMP2 | 6072 |
| SPP1 | 3624 |
| COL1A1 | 3288 |
| ESR1 | 2870 |
| ERBB2 | 2797 |
| PPARG | 2743 |
| SERPINE1 | 2736 |
| VCAM1 | 2382 |
| JAK2 | 1860 |
| RUNX2 | 1519 |
| BCL2L1 | 1236 |
| PLAU | 985 |
| PDGFRB | 984 |
| MCL1 | 978 |
| COL1A2 | 912 |
| CAT | 486 |
| CYR61 | 480 |
| COL3A1 | 408 |
| COL2A1 | 360 |


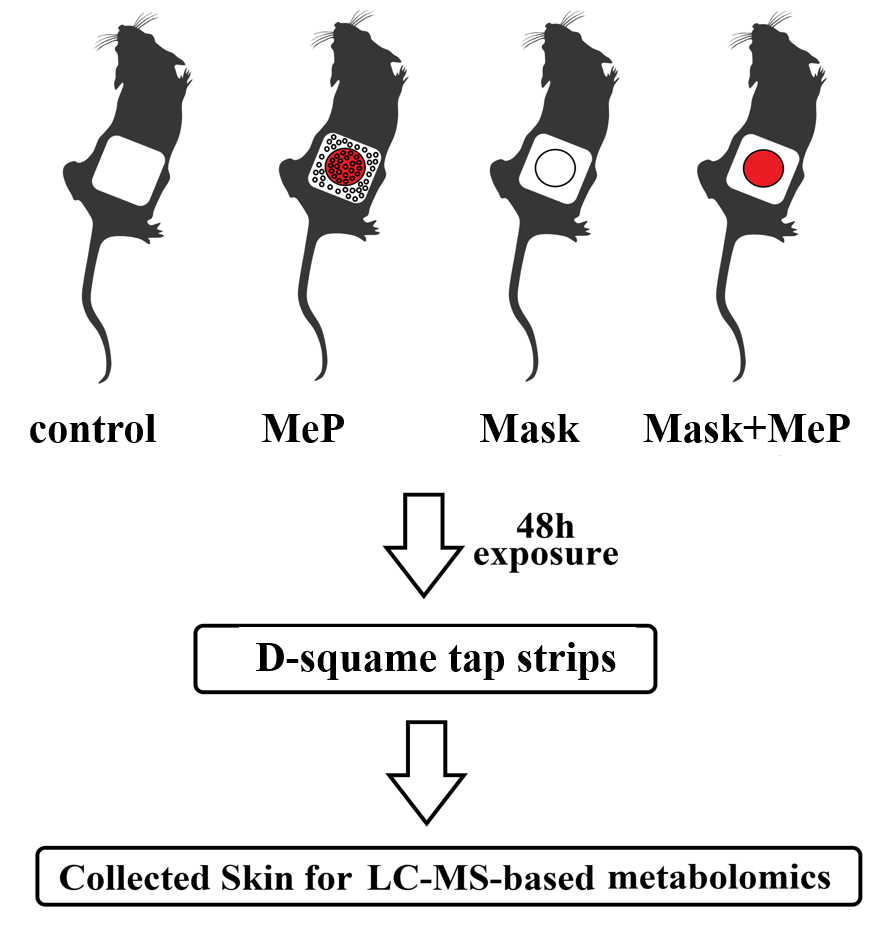


**Figure S1.** Experimental protocol for ICR mice skin exposure.


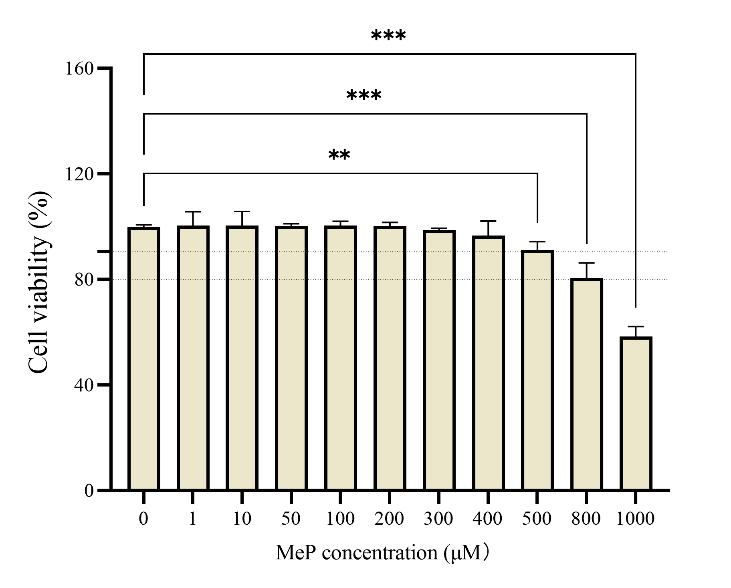


**Figure S2.** Cell viabilities of HaCaT cells under different MeP concentrations. Data were expressed as means ± SD (n = 6). *, ** and *** represent *P* < 0.05, *P* < 0.01, and *P* < 0.001, respectively.


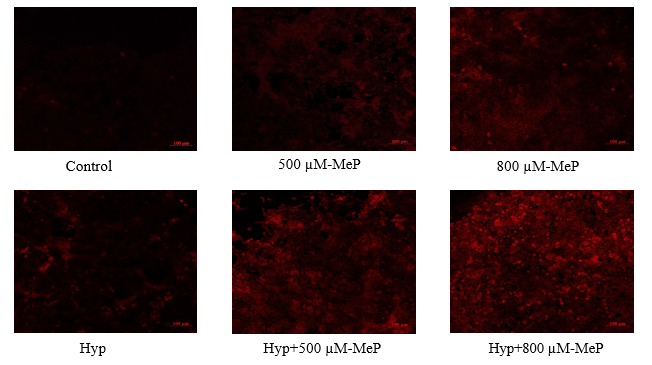


**Figure S3**. Confocal images of ROS in the HaCaT cell after MeP and/or hypoxia treatment.


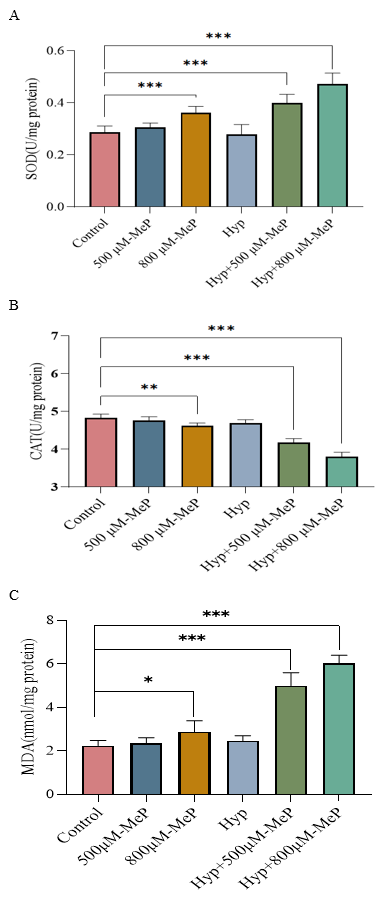


**Figure S4**. The activity of SOD (A) and CAT (B) in HaCaT cells after MeP and hypoxia exposure. The level of MDA in HaCaT cells after MeP and hypoxia exposure. **P* < 0.05, ***P* < 0.01, ****P* < 0.001.


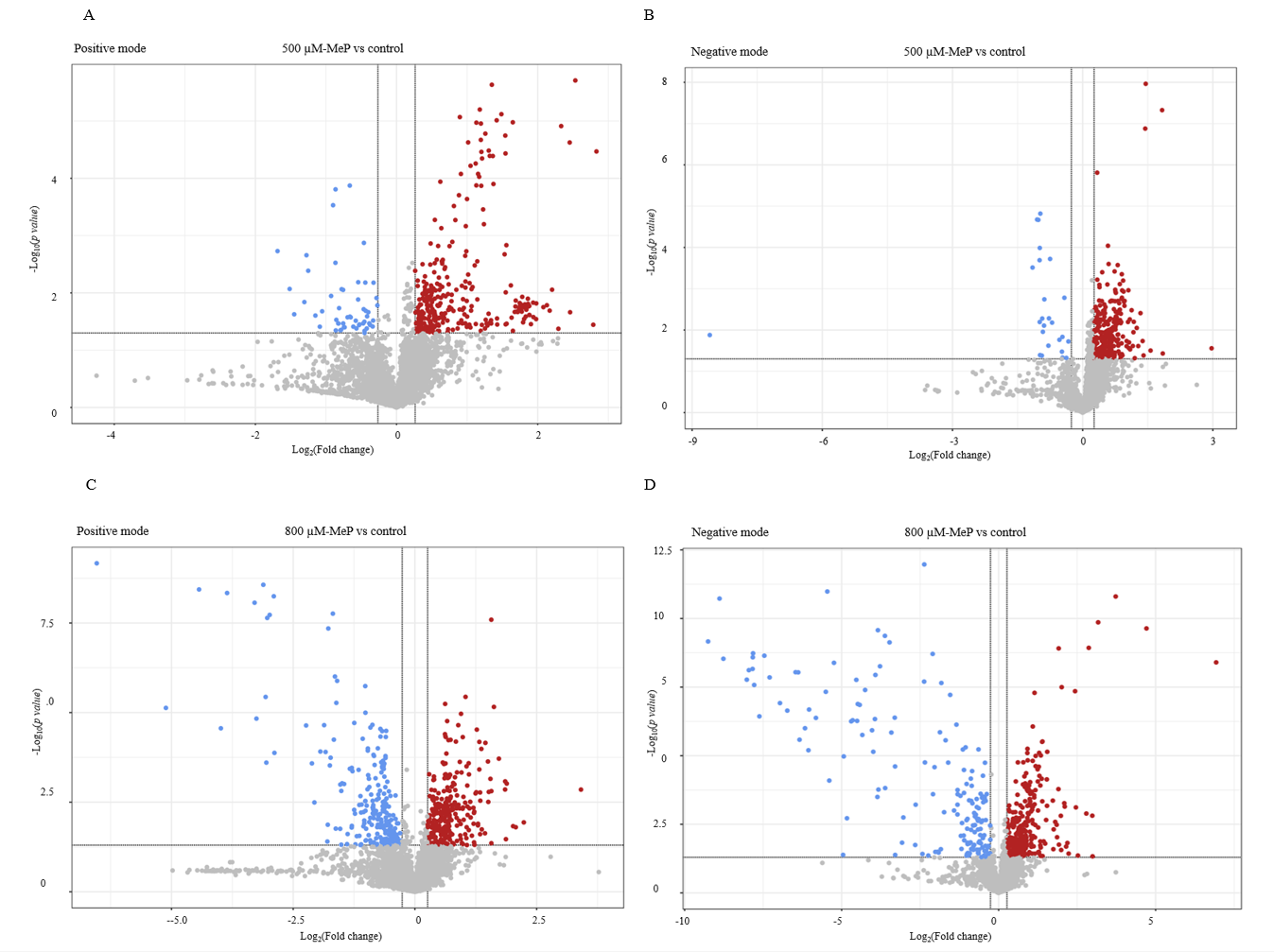


**Figure S5.** Molecular feature volcano plots of the group of "500 μM-MeP" *vs.* "control" in positive mode (A) and negative mode (B), as well as that of "800 μM-MeP" *vs.* "control" in positive mode (C) and negative mode (D). Each point represents a feature. The blue dot represents FC < 0.8 and *P* < 0.05, and the red square represents FC > 1.2 and *P* < 0.05.


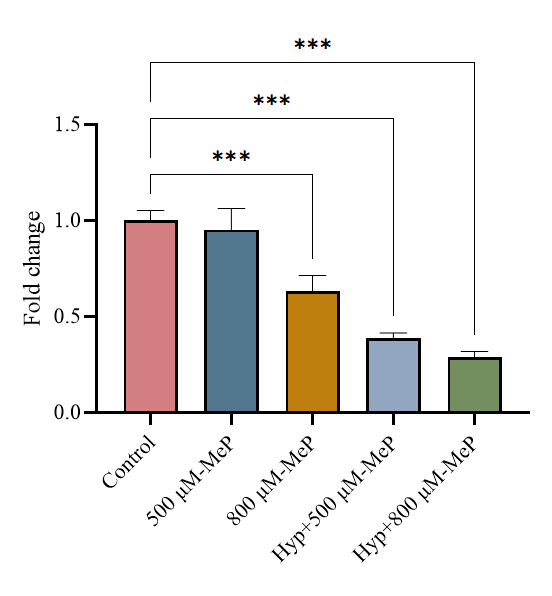


**Figure S6.** Fold change of GSH/GSSG in HaCaT cells after different MeP concentrations and/or hypoxia treatment. *** *P* < 0.001.


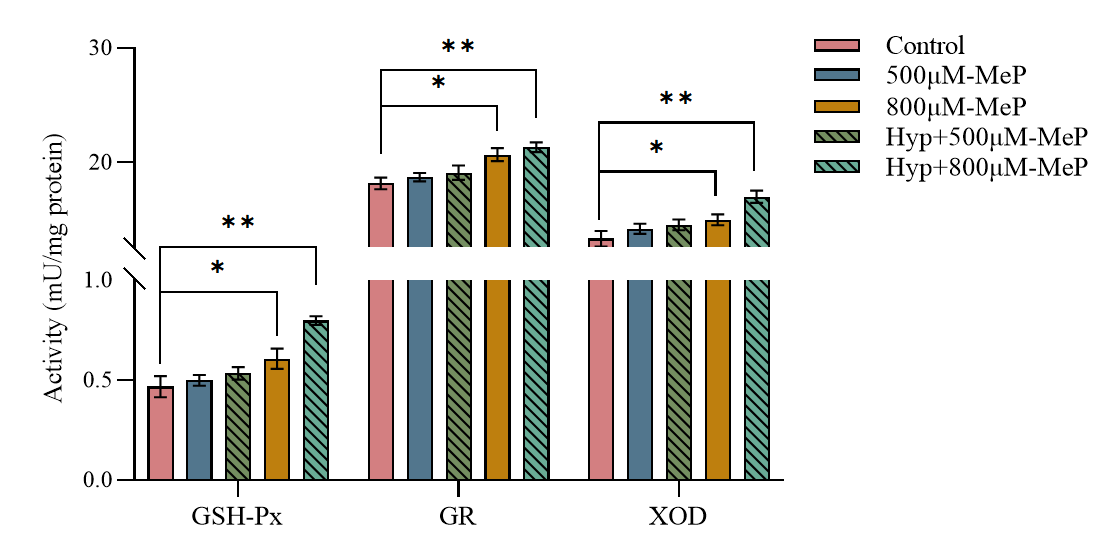


**Figure S7.** Activities of GSH-Px, GR and XOD in HaCaT cells after different MeP concentrations and/or hypoxia treatment. **P* < 0.05, ***P* < 0.01.


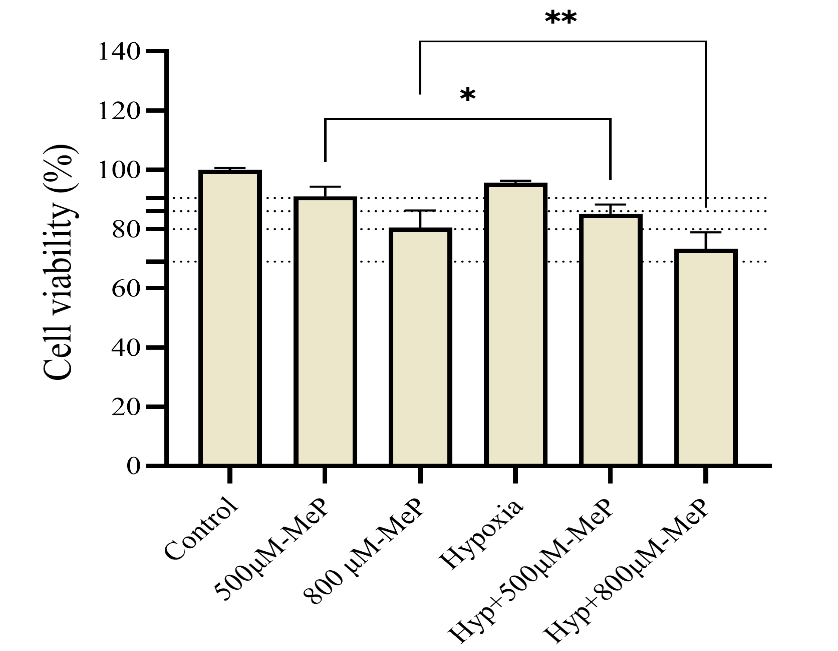


**Figure S8.** Cell viabilities of HaCaT cells under hypoxia and/or MeP treatment. Data were expressed as means ± SD (n = 6), * and ** represent *P* < 0.05 and *P* < 0.01.


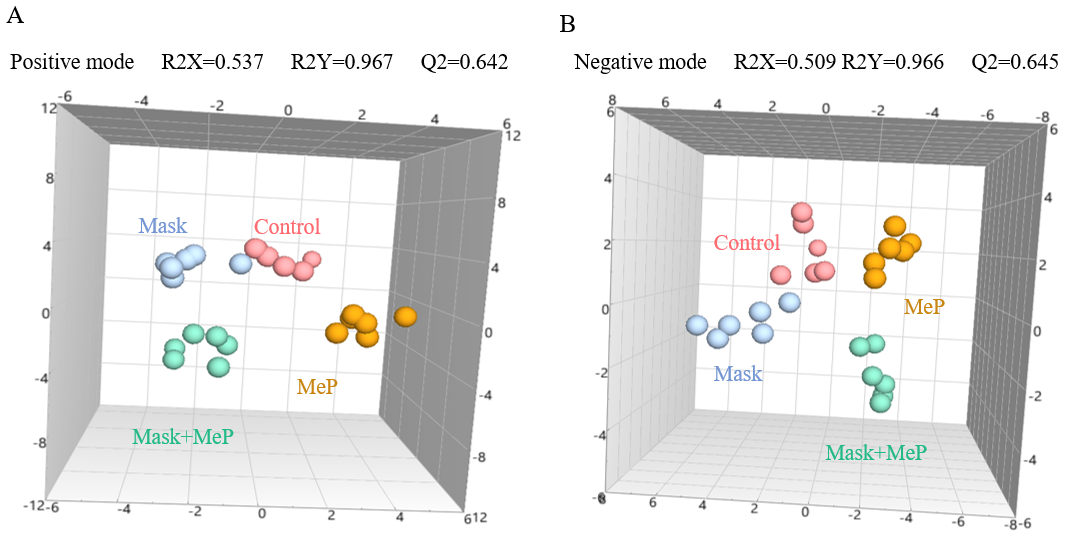


**Figure S9.** Three-dimensional PLS-DA score plots of mice skin extract of the control group, Mask group, MeP group, and Mask+MeP group in positive (A) and negative (B) mode.


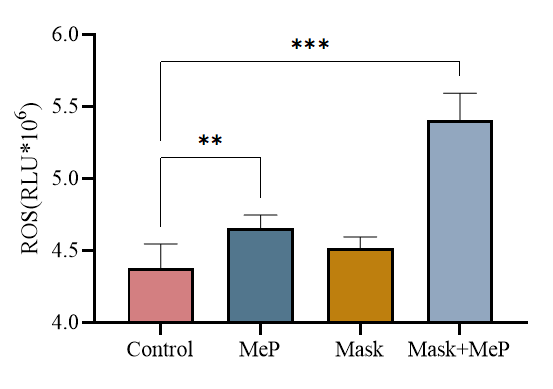


**Figure S10.** The level of ROS in mice skin. Data were expressed as means ± SD (n = 6), ** and *** represent *P* < 0.01 and *P* < 0.001, respectively.


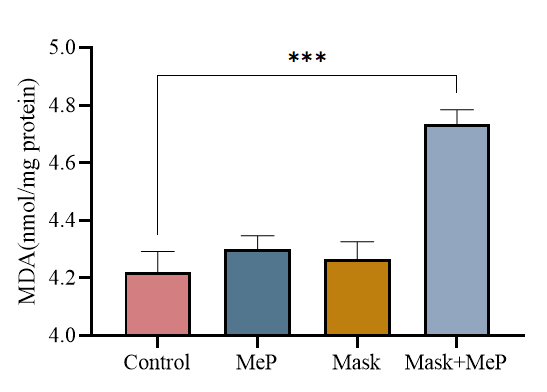


**Figure S11.** The level of MDA in mice skin. Data were expressed as means ± SD (n = 6), ****P* < 0.001.


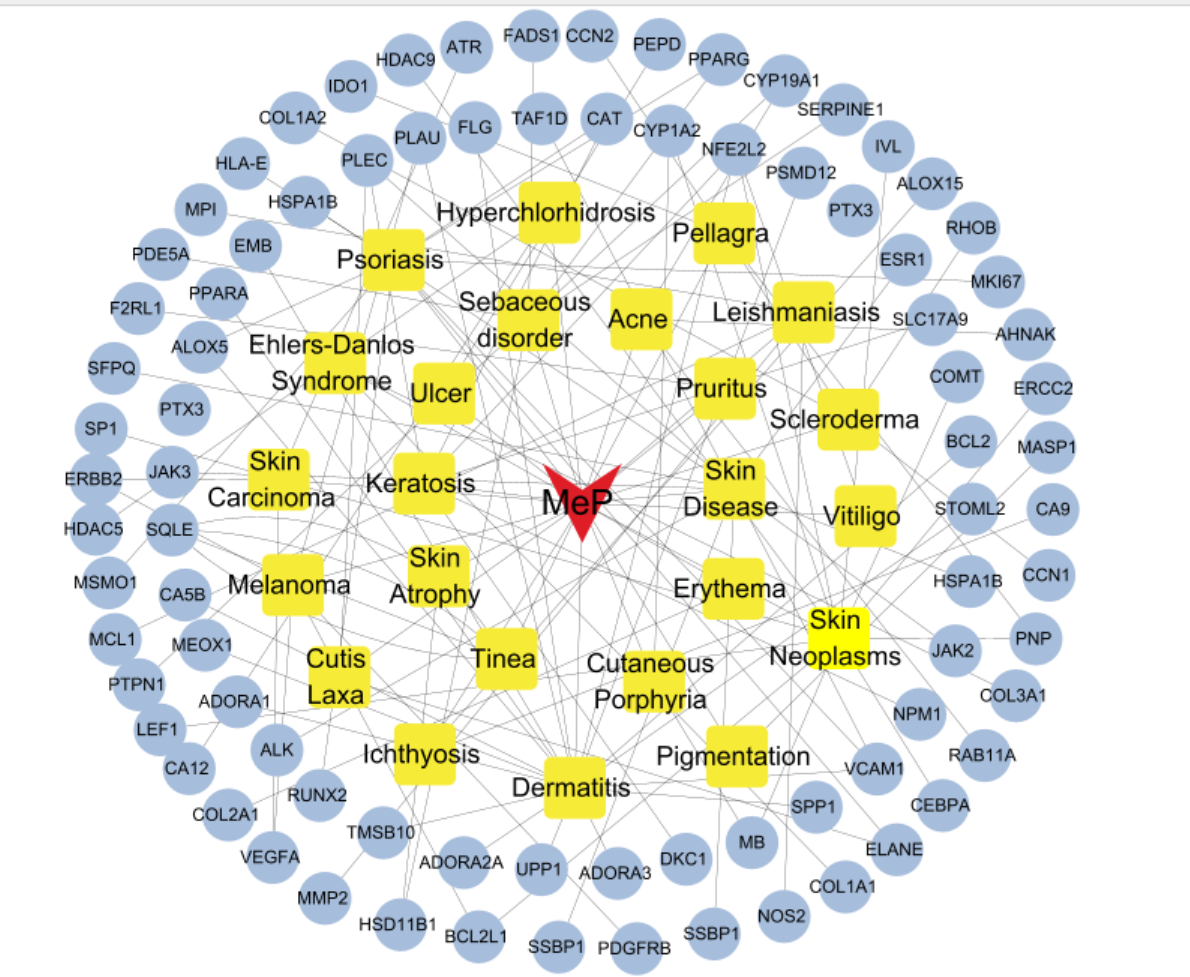


**Figure S12.** “Chemical-targets-risk” network of MeP based on the network toxicology analysis. The blue nodes represent target genes, the yellow nodes represent diseases, and the red nodes represent MeP.


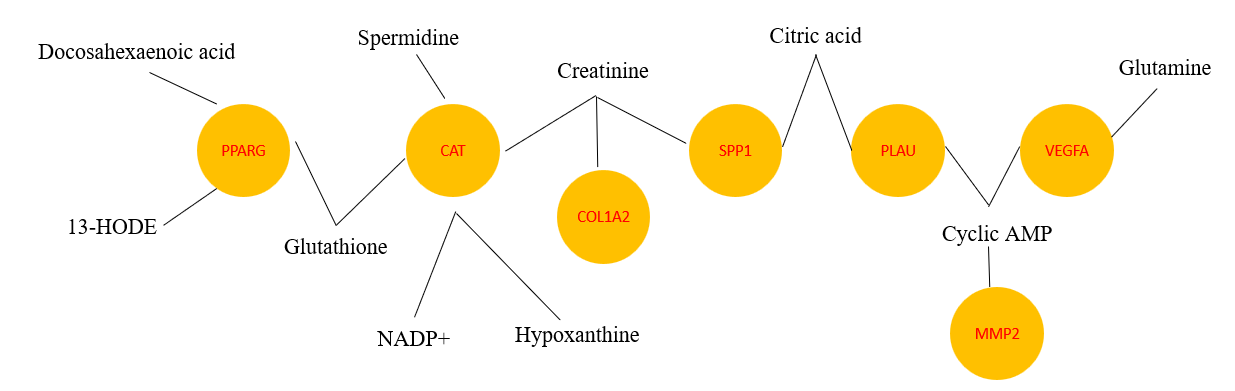


**Figure S13.** The relationship between VEGFA, MMP2, SPP1, ERBB2, PPARG, JAK2, PLAU, PDGFRB, COL1A2 and CAT with differential metabolites, as cited from MetaboAnalyst (version

5.0, htttp://https://www.metaboanalyst.ca/).
